# Systematic review of influenza resistance to the neuraminidase inhibitors

**DOI:** 10.1186/1471-2334-11-134

**Published:** 2011-05-19

**Authors:** Kristian Thorlund, Tahany Awad, Guy Boivin, Lehana Thabane

**Affiliations:** 1 Department of Clinical Epidemiology and Biostatistics, Faculty of Health Sciences, McMaster University, Hamilton, L8N 3Z5 Ontario, Canada; 2 Centre de recherche en infectiologie, CHUQ-CHUL, 2705 boul. Laurier, Québec (Québec), Canada, G1V 4G2, Canada; 3 Biostatistics Unit, Father Sean O'Sullivan Research Centre, St Joseph's Healthcare - Hamilton, Hamilton L8N 4A6 Ontario, Canada

## Abstract

**Background:**

Antivirals play a critical role in the prevention and the management of influenza. One class of antivirals, neuraminidase inhibitors (NAIs), is effective against all human influenza viruses. Currently there are two NAI drugs which are licensed worldwide: oseltamivir (Tamiflu^®^) and zanamivir (Relenza^®^); and two drugs which have received recent approval in Japan: peramivir and laninamivir. Until recently, the prevalence of antiviral resistance has been relatively low. However, almost all seasonal H1N1 strains that circulated in 2008-09 were resistant to oseltamivir whereas about 1% of tested 2009 pandemic H1N1 viruses were found to be resistant to oseltamivir. To date, no studies have demonstrated widespread resistance to zanamivir. It seems likely that the literature on antiviral resistance associated with oseltamivir as well as zanamivir is now sufficiently comprehensive to warrant a systematic review.

The primary objectives were to systematically review the literature to determine the incidence of resistance to oseltamivir, zanamivir, and peramivir in different population groups as well as assess the clinical consequences of antiviral resistance.

**Methods:**

We searched MEDLINE and EMBASE without language restrictions in September 2010 to identify studies reporting incidence of resistance to oseltamivir, zanamivir, and peramivir. We used forest plots and meta-analysis of incidence of antiviral resistance associated with the three NAIs. Subgroup analyses were done across a number of population groups. Meta-analysis was also performed to evaluate associations between antiviral resistance and clinical complications and symptoms.

**Results:**

We identified 19 studies reporting incidence of antiviral resistance. Meta-analysis of 15 studies yielded a pooled incidence rate for oseltamivir resistance of 2.6% (95%CI 0.7% to 5.5%). The incidence rate for all zanamivir resistance studies was 0%. Only one study measured incidence of antiviral resistance among subjects given peramivir and was reported to be 0%. Subgroup analyses detected higher incidence rates among influenza A patients, especially for H1N1 subtype influenza. Considerable heterogeneity between studies precluded definite inferences about subgroup results for immunocompromised patients, in-patients, and children. A meta-analysis of 4 studies reporting association between oseltamivir-resistance and pneumonia yielded a statistically significant risk ratio of 4.2 (95% CI 1.3 to 13.1, p = 0.02). Oseltamivir-resistance was not statistically significantly associated with other clinical complications and symptoms.

**Conclusion:**

Our results demonstrate that that a substantial number of patients may become oseltamivir-resistant as a result of oseltamivir use, and that oseltamivir resistance may be significantly associated with pneumonia. In contrast, zanamivir resistance has been rarely reported to date.

## Background

### Description of the Condition

Influenza (the flu) is an acute infection of the upper respiratory tract which is transmitted via respiratory droplets and direct contact. Immunocompromised people and those with underlying cardio-pulmonary conditions are considered at increased risk from serious influenza-related complications. Annually, influenza infection results in more than 500, 000 deaths worldwide [1]. The influenza virus is an RNA virus that belongs to the Orthomyxoviridae family [2]. There are two main types of influenza virus: type A and type B [2]. These two types are responsible for seasonal flu epidemics each year. The influenza virus is continually evolving and under immune pressure; it may either evolve through small gradual changes in the virus (antigenic drift) or through abrupt major changes in the virus (antigenic shift) most frequently by genetic reassortments [3]. Such changes can result in the emergence of new influenza viruses that can cause pandemics (e.g., the 1918 Spanish flu pandemic and the 2009 H1N1 pandemic)[4].

### Description of the interventions

Vaccines play a critical role in the prevention of influenza [5]. Nevertheless, the efficacy of this intervention could be significantly reduced owing to a mismatch between the seasonal influenza vaccine and the circulating influenza virus and the inability of the host to mount a proper immune response [6]. Therefore, antivirals also play an important role in the prevention and management of influenza. There are two classes of antiviral agents for influenza: adamantanes and neuraminidase inhibitors. Adamantanes (amantadine and rimantadine), however, are not recommended alone for the treatment of influenza due to their lack of activity against influenza B and the high level of influenza A resistance [6,7]. Neuraminidase inhibitors (NAIs) are effective against all human, avian and animal influenza viruses [6-8]. NAIs inhibit the release of virions by competitively inhibiting viral NA, which is a key glycoprotein at the surface of the virus. Currently there are two NAIs drugs which have been approved worldwide: Oseltamivir (Tamiflu^®^) and Zanamivir (Relenza^®^). Both drugs are approved for treatment of acute uncomplicated illness due to influenza A and B, and are also approved for preventive use [9]. Oseltamivir is provided orally to persons older than one year who have been symptomatic for no more than 2 days [9]. Zanamivir is provided as a dry powder which is given by inhalation in persons aged 7 years and older who have been symptomatic for no more than 2 days [9].

Peramivir is an intravenous neuraminidase inhibitor under development for the treatment of influenza. In October 2009, the FDA issued an Emergency Use Authorization (EUA) for the use of peramivir based on safety data from Phase 1, Phase 2 trials, and limited Phase 3 trial data. The EUA for peramivir expired in June 2010. However, peramivir has been approved in Japan and Korea. More recently, laninamivir, a long-lasting inhaled neuraminidase inhibitor has also been licensed in Japan [10,11].

### Antiviral resistance

Influenza A and B strains had remained susceptible to oseltamivir with rare exceptions since their availability in 1999. Recent reviews and expert opinions have identified studies reporting prevalence and incidence rates of antiviral resistance among patients treated with oseltamivir [6,7]. Until recently, the prevalence of antiviral resistance had been relatively low [12,13]. However, in the influenza season 2008-2009, the Centers for Disease Control and Prevention (CDC) reported a worldwide significant increase in the prevalence of oseltamivir resistance to influenza A/H1N1 viruses (A/Brisbane/59/2007-like strains) due to the H275Y NA mutation [9]. Furthermore, about 1% of tested 2009 pandemic H1N1 viruses (A/California/07/2009 (H1N1) - like strain) were found to be resistant to oseltamivir due to the same mutation. Finally, a Japanese study published in 2004 reported incidence of antiviral resistance in hospitalized children as high as 18%[14].

To date influenza A and B, including all types of A/H1N1 virus, remain susceptible to zanamivir. Further, a recent review only found one documented case of zanamivir resistance [6,15]. Oseltamivir is easier to administer and has therefore been used vastly more than zanamivir. For this reason, most literature on antiviral resistance associated with NAIs has only investigated antiviral resistance associated with oseltamivir. However, owing to the higher resistance rates associated with oseltamivir, especially for A/H1N1 strains between 2007-09, zanamivir has been increasingly utilized for the treatment and prevention of influenza [9]. Therefore, there is a need to investigate the development of influenza resistance to zanamivir in this time period. Studies investigating resistance to peramivir may also have emerged.

We therefore conducted a systematic review using comprehensive literature search strategies to identify and systematically assess all relevant studies on antiviral resistance associated with oseltamivir, zanamivir, and peramivir.

## Objectives

### Primary objective

Our primary objective was to systematically review the literature to determine the incidence of resistance to oseltamivir, zanamivir, and peramivir in adult immunocompetent outpatients for the prevention and treatment of influenza. We did not consider lananimivir since it was not licensed at the time we commenced this review.

### Secondary objectives

Our secondary objectives were to

1) Systematically review data from the literature to determine the incidence of resistance to oseltamivir, zanamivir, and peramivir in different patient populations (e.g., hospitalized, immunocompromised, children, and others).

2) Assess the clinical consequences of antiviral resistance (clinical complications and symptoms associated with antiviral resistance, and severity of complications and symptoms among resistant patients).

## Methods

### Criteria for considering studies for this review

#### Types of studies

We included randomized clinical trials (RCTs), cohort and case-control studies, and case reports.

#### Types of patients

We included adult immunocompetent outpatients for our primary objective. For our secondary objective we included patients if they were children, immunocompromised, or hospitalized.

#### Types of interventions

We included studies that reported incidence of antiviral resistance associated with one or more of the three NAIs oseltamivir, zanamivir, and peramivir. We included all studies regardless of the dose and length of follow-up. We did not include studies where an NAI was administered in combination with another antiviral (e.g., rimantadine)

#### Types of outcomes

Our primary and first secondary outcome of interest was incidence of antiviral resistance and our secondary outcomes of interest were the clinical consequences associated with antiviral resistance.

Viral resistance is detected either by the presence of resistance-associated mutations such as the H274Y (N2 numbering) or H275Y (N1 numbering) substitution or by measuring the median 50% inhibitory concentration (IC_50_). Commonly used laboratory criteria for determining known antiviral resistance is a substantially elevated IC_50 _value by enzyme inhibition assay (a value greater than 10-fold that of the corresponding parent virus or greater than three standard deviation (SD) compared to the mean value [13,16]. For example, for the H1N1 virus with the most frequent H275Y NA mutation, the observed IC50 values are usually 200- 400 fold higher than the mean IC50 value [17-21]. However, the definition of antiviral resistance is not standardized and could differ for each of the three NAIs, and may also vary across included studies (due to pre-specification of a threshold or different assays). For that reason, we used the definition of resistance as outlined in the studies to determine the incidence of antiviral resistance.

Measured influenza outcomes included duration of antiviral shedding, the peak viral titers, and the days to resolution of influenza symptoms. For studies reporting on clinical consequences of antiviral resistance, we reported all influenza related complications and symptoms.

### Electronic searches and data retrieval

We searched MEDLINE and EMBASE without language restrictions in September 2010. We limited the search strategy to human studies. We also manually searched reference lists from recent review articles.

#### Study selection and Data Extraction

Two reviewers (KT and TA) independently reviewed the abstracts for potential eligibility and subsequently full text publications for eligibility. Disagreements were resolved by discussion.

We extracted a number of variables on study design and methodological characteristics, patient and intervention characteristics, and outcomes from all eligible studies (see appendix). Data extraction was done independently by two reviewers (KT and TA) and disagreements were resolved by discussion.

### Methodological Quality Assessment

For the RCTs, we assessed the adequacy of the methods used for randomization, allocation concealment, blinding, and follow-up [22,23]. We scored the quality for each RCT by assigning one point for each adequate item. With this scale, RCTs can score a minimum of 0 points and a maximum score 4 points. We considered RCTs that scored at least 3 points as high quality. We used the Newcastle-Ottawa Scale (NOS) to assess the adequacy of the employed methodology in the retrieved observational studies [24]. For observational studies reporting incidence of antiviral resistance, there were no control groups. We therefore used a modified version of the NOS scale for cohort studies, taking out the three items that dealt with adequacy of controls or comparison between exposed and non-exposed individuals. With this scale, incidence studies can score a minimum of 0 points and a maximum of 6 points. We considered studies that scored at least 5 points as high quality. For observational studies reporting on consequences of antiviral resistance, we used the complete NOS scale. With this scale, incidence studies can score a minimum of 0 points and a maximum of 9 points. We considered studies that scored at least 7 points as high quality.

### Statistical analysis and reporting of results

We calculated the antiviral resistance incidence rate and the associated 95% confidence interval (CI) for all studies and study subgroups. Antiviral resistance incidence rate was calculated as the proportion of patients who developed resistance. We calculated the 95% CI based on the assumption that the incidence rate followed a binomial distribution. For incidence rates equal to 0%, we used the "rule of threes" to calculate the upper 95% CI upper bound [25]. This rule says that the 95% CI upper bound is equal to 3/n, where n is the sample size.

### Primary objective - estimating incidence

We calculated the antiviral resistance incidence rate and the associated 95% CI for all studies (and subgroups) involving immunocompetent adult out-patients. We produced forest plots and pooled incidence rates across studies using the arcsine method [26].

### Secondary objective - incidence across subgroups

We first performed subgroup analysis on type of NAIs (oseltamivir, zanamivir, or peramivir) and pooled incidence rates within each subgroup using the arcsine method [26]. For each type of NAIs, we then performed subgroup analyses for study design, type of influenza, immunocompetent/immunocompromised patients, out patients/hospitalized patients, adults/children, and prophylaxis/treatment and produced corresponding forest plots. We did not produce pooled estimates in these subgroups.

### Secondary objective - consequences of antiviral resistance

We qualitatively compared the mean, median, minimum, and maximum duration of antiviral shedding, the peak viral titers, and the days to resolution of influenza symptoms reported in the identified incidence studies.

We reported odds ratios and risk ratios for associations between antiviral resistance and clinical complications or symptoms. Whenever possible, these ratios were combined in a random-effects meta-analysis and presented in a forest plot. Adjusted ratios obtained from multivariate models were preferred to crude ratios. If possible, risk ratios were converted to odds ratios.

For resistant patients reported in case reports, we created a qualitative overview (a table) of clinical complications (e.g., pneumonia) and symptoms which authors believed could be associated with resistance.

## Results

**Figure 1:**
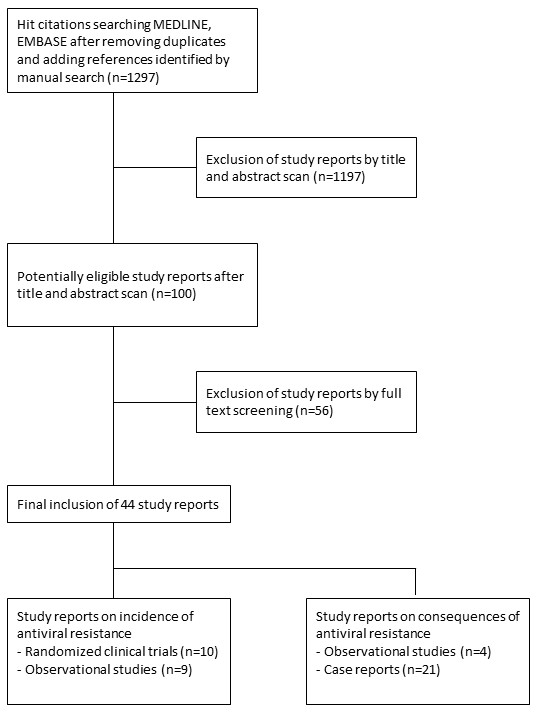
**PRISMA diagram of literature search and eligibility scan**. Study reports were excluded if 1) they were expert opinions, commentaries, letters or reviews; 2) if it was clear that they did not include either oseltamivir, zanamivir, or peramivir treated patients; or 3) if it was clear that none of the considered outcomes could be extracted.

Our search resulted in 1289 hits after removal of duplicate citations. Figure 1 presents the PRISMA diagram for our eligibility scan. A total of 43 study reports were eligible. Among these, ten were randomized clinical trials (RCTs) that reported incidence of antiviral resistance,[27-36] (effectively fourteen since two publications reported results from four and three RCTs respectively) and nine were cohort studies that reported incidence of antiviral resistance [14,37-44]. Four were case-control studies that reported associations between antiviral resistance and clinical symptoms or complications [45-48]. Lastly, 21 were case reports comprising reports on a total of 27 patients [15,49-68]. None of the ten RCTs or the nine cohort studies made head to head comparisons between any two of the three NAIs (i.e., all 19 studies only included one NAI arm). Thirteen studies measured incidence of antiviral resistance among subjects given oseltamivir,[14,31,33,35,37-44], five studies measured incidence of antiviral resistance among subjects given zanamivir,[27,29,30,32,34] and only one study measured incidence of antiviral resistance among subjects given peramivir [28]. Four oseltamivir studies and all zanamivir and peramivir studies were RCTs. The four association studies all estimated associations between oseltamivir resistance and clinical complications and symptoms. Among the 21 case reports, 17 reported on resistance to oseltamivir only, and 2 on resistance to zanamivir only, and 2 on resistance to both. In 12 of the 21 case reports (13 out of 27 patients) where oseltamivir was the initial treatment, patients were eventually switched to zanamivir.

Most studies that reported incidence of antiviral resistance included a mix of our pre-specified subgroups. The methodological quality, the patient characteristics, and the intervention characteristics of the incidence studies are presented in table 1. All RCTs were of high quality. Only one incidence study was low quality according to the modified NOS. The four case-control studies that reported associations between antiviral resistance and clinical symptoms and complications all scored between 7/9 and 9/9 on the NOS.

**Table 1:** Baseline characteristics of studies reporting on incidence of resistance

		Study		Intervention			Patients		
				
Study first author	Recruitment Period	Design*	Quality score**	Purpose	Dose	Duration	Immunostatus	In/ Out	Age group
***Zanamivir studies***									
Boivin^31^	1997-1998	RCT	High	Treatment	10 mg td	5 days	Competent	Out	Unclear
Hedrick^36^	1998-1999	RCT	High	Treatment	10 mg td	5 days	Competent	Out	Children
Ambrozaitis^29^	1997-2000	RCT	High	Prophylactic	10 mg td	14 days	Competent	In	Adults
Gravenstein^32^	1997-2000	RCT	High	Prophylactic	10 mg td	14 days	Competent	In	Adults
Hayden(1)^34^	1999-2000	RCT	High	Prophylactic	10 mg td	10 days	Competent	Out	Adults
***Peramivir study***									
Barosso(a)^30^	1999-2000	RCT	High	Treatment	100, 200, or 400 mg qd, or 200 md q12 h	5 days	Competent	Out	Adults
Barosso(b)^30^	1999-2000	RCT	High	Treatment	800 mg qd(day1) then400 mg q24 or 400 mg q24	5 days	Competent	Out	Adults
Barosso(c)^30^	1999-2000	RCT	High	Treatment	50, 200, or 400 mg qd	5 days	Competent	Out	Adults
Barosso(d)^30^	1999-2000	RCT	High	Treatment	200, 400 or 800 mg	5 days	Competent	Out	Adults
***Oseltamivir studies***									
Whitley^38^	1998-1999	RCT	High	Treatment	2 mg/kg twice daily (max 100 mg/day)	5 days	Competent	Out	Children
Hayden(2)^35^	2000-2001	RCT	High	Prophylactic	75 mg once daily	10 days	Competent	Out	Mix
Hayden(3-a)^33^	2000	RCT	High	Treatment	75 mg/150 mg once/twice daily	5 days	Competent	Out	Adult
Hayden (3-b)^33^	2000	RCT	High	Treatment	75 mg once daily	5 days	Competent	Out	Adult
Hayden (3-c)^33^	2000	RCT	High	Prophylactic	75 mg once daily	7 days	Competent	Out	Adult
Ison^37^	Before 2009	RCT	High	Prophylactic	75 mg orally or suspension once daily for age = > 13, weight-based or suspension for age < 13	12 days	Compromised	In	Unclear
Kiso^15^	2002-2003	OBS	6/6	Treatment	4 mg/kg	2-5 days	Competent	Mix	Children
Kawai^42^	2003-2004	OBS	6/6	Treatment	75 mg twice daily for adults and children > 35 kg, weight-based twice daily for children < 35 kg	5 days	Unclear	Out	Mix
Hatekayama^41^	2004-2005	OBS	5/6	Treatment	Unclear	Unclear	Mix	Unclear	Children
Stephenson^43^	2005-2007	OBS	6/6	Treatment	Twice daily weight-based dosing regimen	5 days	Competent	Unclear	Mix
Cost^39^	2009	OBS	4/6	Prophylactic	Unclear	Unclear	Compromised	In	Children
Harvala^40^	2009	OBS	6/6	Treatment	Unclear	Unclear	Mix	Unclear	Unclear
Tramontana^44^	2009	OBS	6/6	Treatment	Varied across patients	Up to 43 days	Compromised	In	Adults
Wang^45^	2009	OBS	6/6	Treatment	Unclear	Unclear	Mix	In	mix
Winzer^46^	2009	OBS	5/6	Treatment	Unclear	Unclear	Unclear	Unclear	mix

*Study design is either a randomized clinical trial (RCT) or an observational study (OBS)

**Quality scores for RCTs range from 1 to 4. Study scores for OBSs range from 1 to 6. Low scores indicate low quality, high scores indicate high quality.

### Primary objective - estimating incidence

Most studies included a mix of the considered subgroups but did report results separately for each subgroup. It was therefore only possible to extract data from five studies for our primary objective: incidence of resistance among adult immunocompetent out-patients [27-31]. Among these five studies, one reported incidence of antiviral resistance associated with oseltamivir, three reported incidences of antiviral resistance associated with zanamivir, and one reported incidences of antiviral resistance associated with peramivir. In all five studies, patient recruitment ended in or before year 2000. Incidence of resistance was 0% in all five studies [27-31]. For this reason we did not produce a forest plot.

### Secondary objective - incidence across subgroups

**Figure 2:**
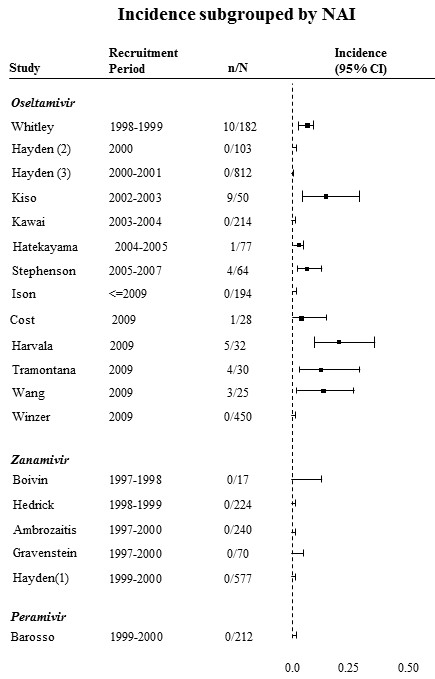
**Forest plots of antiviral resistance incidence among oseltamivir studies subgrouped by neuraminidase inhibitor**. The numerator is the number of patients who developed resistance, and the denominator is the number of patients that received an NAI.

Figure 2 shows the incidence of antiviral resistance subgrouped by NAI (oseltamivir, zanamivir, and peramivir). The pooled incidence rate for oseltamivir was 2.6% (95%CI 0.7% to 5.5%). However, the estimated heterogeneity was I^2 ^= 90%. The incidence rate for all zanamivir studies was 0%. All zanamivir studies were RCTs. Further, all zanamivir studies ended in or before year 2000. Only one study measured incidence of antiviral resistance among subjects given peramivir and was reported to be 0%.

**Figure 3:**
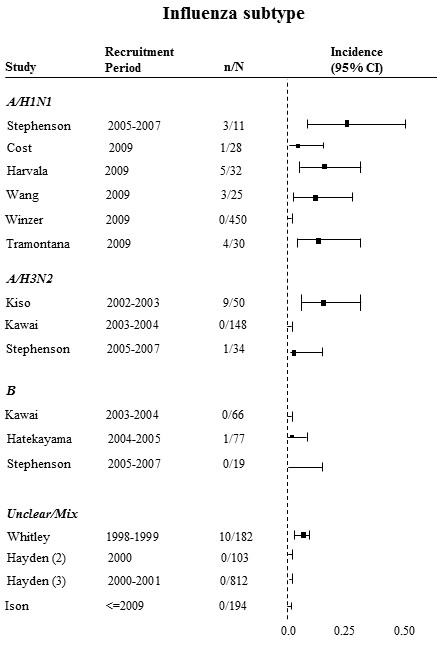
**Forest plots of antiviral resistance incidence among oseltamivir studies subgrouped by type of influenza**. The numerator is the number of patients who developed resistance, and the denominator is the number of patients that received an NAI.

Because antiviral resistance was only observed in oseltamivir studies, we did not produce subgroup forest plots including zanamivir or peramivir studies. Figure 3 presents the forest plot of incidence of antiviral resistance to oseltamivir subgrouped by influenza type. The remaining subgroup forest plots of incidence of antiviral resistance are presented in additional files 1, 2, 3, 4 and 5 (figures A.1 to A.5).

Antiviral resistance was observed among A/H1N1 patients in 5 of 6 studies with incidence rates varying from 3.6% and 27.3%. Among influenza A/H3N2 patients, incidence rates across three studies were 0%, 3.3%, 18%. Incidence rates among influenza B patients were 0% in two studies and 2% in one study (see figure 3). Antiviral resistance rates in prophylaxis studies were either 0% or low (4%), whereas more than half of the treatment studies reported resistance rates above 5% (see additional file 1, figure A.1).

Our subgroup analyses on RCTs versus observational studies, immunocompetent versus immunocompromised, out-patients versus in-patients, and adults versus children did not yield any apparent differences between subgroups. The lack of apparent differences was, in part, due to the large degree of heterogeneity within subgroups. All remaining subgroup analyses are presented in the appendices (see additional files 2, 3, 4 and 5 (figures A.2-A.5)).

### Secondary objective - consequences of antiviral resistance

**Figure 4:**
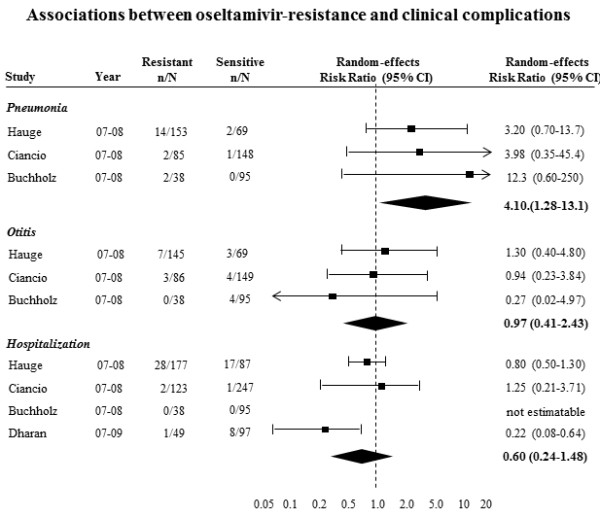
**Forest plots of risk ratios for associations between antiviral resistance and clinical complications**. The risk ratios from the studies by Hauge and Ciancio are adjusted risk ratios obtained from multivariable models. The risk ratios from the Buchholz study are crude risk ratios based on zero-event continuity correction of 0.5. The Dharan risk ratio was transformed from an adjusted odds ratio based on a multivariable model.

Figure 4 shows the forest plot of risk ratios for association between oseltamivir resistance and clinical complications that were reported in more than one study. The meta-analysis of the association between oseltamivir-resistance and pneumonia yielded a statistically significant risk ratio (RR 4.16, 95% CI 1.28 to 13.1, p = 0.02). The meta-analyses of the association with otitis yielded a pooled risk ratio of 0.97 (95% CI 0.41 to 2.43). The meta-analysis of the association with hospitalization yielded a pooled risk ratio of 0.60 (95% CI 0.24 to 1.48). The study by Hauge additionally reported adjusted risk ratios for the associations with sinusitis (RR 1.7, 95% CI 0.4 to 7.5) and with bronchitis (RR 0.8, 95% CI 0.4 to 1.8)[47]. Two deaths were reported in the study by Dharan [48]. However, these two patients died before they were admitted to the hospital. No deaths were reported in the other three identified association studies.

The meta-analyses of associations between oseltamivir resistance and the clinical symptoms (cough, fever, sore throat, myalgia, acute/sudden onset, runny nose and headache), did not reveal any differences between oseltamivir-resistant and oseltamivir-susceptible patients. All pooled risk ratio estimates were equal or very close to 1.00 and all 95% CIs included 1.00. The forest plots for these meta-analyses are presented in additional file 6 and 7 (figure A.6). One study (Dharan) additionally reported odds ratios on chills and breathing difficulty [48]. Risk ratio estimates for these two symptoms were close to 1.00 and had wide confidence intervals.

Table 2 provides a summary of the duration of illness reported across association and incidence studies. Overall, the oseltamivir-resistant and oseltamivir-susceptible patients seem to require the same number of days to reach resolution of symptoms.

**Table 2:** Summary of symptoms and days to resolution of symptoms reported across association studies and incidence studies

Study	Resistant patients		Sensitive patients	
	
	n	Reported symptoms and durations	n	Reported symptoms and durations
***Association studies***				
*Buchholz (2009)^48^*	11	Duration of sick leave: Median: 7 days IQR: 6 to 14	26	Duration of sick leave: Median: 7 days IQR: 7 to 10
*Buchholz (2009)^48^*	38	Days confined to bed: Median: 3.5 days IQR: 2 to 7	93	Days confined to bed: Median: 3 days IQR: 2 to 5
*Dharan (2009)^50^*	49	Activities limited: 1-7 days Median: 4 days	97	Activities limited: 1-30 days Median: 4 days
*Dharan (2009)^50^*	38	Missed work or school: 1-10 days Median: 4 days	97	Missed work or school: 1-30 days Median: 4 days
***Incidence studies***				
*Kiso (2004)^15^*	9	Duration of fever: 2-7 days Median = 3 days	41	Duration of fever: 1-6 days Median = 3 days
*Hatakeyama (2007)^41^*	1	Duration of fever: 2 days	76	Mean duration of fever: 2.4 days
*Stephenson (2009)^43^*	4	Duration of fever: 5-6 days Full recovery after: 9-10 days	60	Authors commented no difference observed
				between resistant and sensitive patients

The identified case reports showed a wide spectrum of complications associated with antiviral resistance. Table 3 summarized these complications in relation to the type of treatment, patient group and influenza. The complications associated with antiviral resistance were typically of a respiratory nature. Prolonged fever was also common. In 9 case reports, oseltamivir resistant patients were switched to zanamivir once viral resistance had been detected. In some cases, patients seemed to recover as a result of the switch, in others cases they did not.

**Table 3:** Summary of patient and treatment characteristics, and complications believed to be associated with antiviral resistance in the reviewed case reports

Study	Period	Flu type	Mutation	Proph/ Treat	Competent/ Compromised	In/Out Patient	Age	Days to resistance detection	Complications associated with resistance
***Oseltamivir resistance***									
Baz^52^	2005-2006	H3N2	E596G E119V I222V I223V	Treat	Compromised	In	4 m	14	Respiratory complications, prolonged viral shedding (about 90 days)
Baz^52^	2009	H1N1	H275Y	Postexp Proph	Competent	Out	59y	8	None. Recovered after 2 weeks
Campanini^55^	2009	H3N2	H274Y	Treat	Compromised	In	2y	18	42 days to resolution of flu symptoms
Cane^56^	2010	H1N1	H275Y	Treat	Compromised	In	3y	NA	1 month to resolution of flu symptoms
CDC-Weekly(1)^57^	2009	H1N1	H275Y I223V	Proph	Competent	Out	Teens	27	None ("uneventful recovery")
CDC-Weekly(1)^57^	2009	H1N1	H275Y I223V	Proph	Competent	Out	Teens	14	None ("uneventful recovery")
CDC-Weekly(2)^48^	2009	H1N1	H275Y	Treat	Compromised	In	Teens	30	2 months to recovery
CDC-Weekly(2)^48^	2009	H1N1	H275Y	Treat	Compromised	In	40y	30	2 months to revovery, prolonged neutron-penia and protracted bone marrow recovery, neutropenic fever, coagulase-negative Staphylococcus bacteremia, and Pneumocystis
Couturier(1)^59^	2009	H1N1	H275Y	Treat	Compromised	In	69y	9	Renal function declined. Died after 17 days.
Couturier(2)^59^	2009	H1N1	H275Y	Treat	Compromised	In	66y	21	Recovered after 1 month. Caught fever again after 3 months and died.
Couturier(3)^59^	2009	H1N1	H275Y	Proph	Competent	Out	36y	NA	None
De Jong^54^	2005	H5N1	H274Y	Treat	Competent	In	13y	5	Patient died. Suffered from hypoxia and pneumonia
Dulek^60^	2009	H1N1	H275Y	Treat	Compromised	In	18 m	22	52 days of viral shedding, progressive pulmonary disease
Gaur^61^	2009	H1N1	H275Y	Treat	Compromised	In	10y	14	None reported
Glikman^62^	2009	H1N1	H275Y	Treat	Compromised	In	11y	13	Prolonged fever (> 21 days)
Hill-Cawthorne^63^	2009	H1N1	H275Y	Treat	Compromised	Out	56y	20	Prolonged fever and cough (2 months)
Ison(1)^64^	2001	NA	Asp198Asn Aer285Ala	Proph	Compromised	In	2y	42	Continued rhinorrhea and dry cough, progressive respitatory distress, gastrointestinal bleeding. Eventually died.
Ison(2)^64^	2002	H3N2	Arg142Gly Tyr195Phe	Treat	Compromised	In	63y	90	Prolonged cough and nasal congestion
Ison(3)^64^	2003	H3N2	Glu119Val Ser31Asn	Treat	Compromised	In	60y	NA	Progressive respiratory compromise and pneumonia, continued respiratory failure. Died of haemorrhagic stroke.
Le^53^	2005	N5N1	H274Y	Both	Competent	Out	14y	4	None. Recovered after 2 weeks.
Memoli^65^	2009	H1N1	Amino-acid position 245-248	Treat	Compromised	In	43y	5	None. Recovered after 2 weeks.
Nguyen**^66^	2009	H1N1	H27Y	Treat	Compromised	In	14y	17	Died of complications after 2 months
			I223R						
Rousset**^67^	2009	H1N1	H275Y	Treat	Compromised	In	24y	6	Patient died at day 140
Speers^68^	2009	H1N1	H275Y	Treat	Compromised	In	38y	3	None. Eventually died
Thabet^69^	2009	H1N1	H274Y	Treat	Compromised	In	3y	10	None reported.
Van der Vries*^70^	2009	H1N1	H275Y	Treat	Compromised	In	5y	7	Resolutions of symptoms after 3 months
***Zanamivir resistance***									
Gubareva^16^	1998	NA	152Argr-Lys	Treat	Compromised	In	18 m	8	Not resolved, patients died.
Van der Vries*^70^	2009	H1N1	I223R	Treat	Compromised	In	5y	56	Resolutions of symptoms after 3 months

* patient was treated with oseltamivir first, zanamivir second, and developed resistance to both antivirals

** patient also had reduced sensitivity to zanamivir

## Discussion

The primary focus of this review was to describe the incidence of resistance to oseltamivir, zanamivir, and peramivir in adult immunocompetent outpatients for the prevention and treatment of influenza. However, we did not identify sufficient evidence to draw any conclusions about this issue. The secondary focus of our review was to describe the incidence and consequences of antiviral resistance across various population groups. Overall the incidence of oseltamivir-resistance across studies was 2%. We found higher oseltamivir-resistance incidence rates among influenza A patients - particularly those of the H1N1 subtype. Zanamivir was associated with 0% incidence of resistance. Only one study measured incidence of antiviral resistance among subjects given peramivir and it was reported to be 0%. However, zanamivir and peramivir clinical trials stopped enrolling in year 2000 at the latest, and therefore have little generalizability. We did not compare the incidence rates between drugs because there are no head-to-head studies that have compared these drugs. Our analyses of consequences associated with development of antiviral resistance demonstrated that oseltamivir-resistant patients are approximately 4 times more likely to suffer from pneumonia than oseltamivir-susceptible patients. We did not find any associations between oseltamivir-resistance and other clinical complications or symptoms. We did not identify any studies reporting on association between zanamivir or peramivir resistance and clinical complications or symptoms. Our review of case reports shows that antiviral resistance can be associated with a number of serious complications. Such complications are typically of a respiratory nature and may in some cases be so severe that the patient will die, but whether it depends on viral resistance or on the host's immune response needs to be determined.

Our review comes with a number of strengths and limitations. We collectively reviewed randomized clinical trials, cohort studies, case series, and case reports. Randomized clinical trials are typically conducted under controlled settings that may not be entirely representative of general clinical practice. In the context of our review, such controlled settings may substantially reduce the risk of developing antiviral resistance. By including observational studies in our review, we obtain incidence estimates that are generalizable to conventional clinical practice settings. Furthermore, our additional inclusion of case reports allowed us to evaluate the severity of the clinical complications that can arise from antiviral resistance.

Despite including a wide range of considered study designs, our review finds its limitations in the paucity of evidence. We did not find any recent studies that investigated incidence of antiviral resistance associated with zanamivir or peramivir, in fact, all studies on zanamivir ended patients' recruitment in 2000 at the latest. Thus, we were unable to assess whether zanamivir and peramivir still yields a (close to) 0% incidence of antiviral resistance. In addition to the time factor, it is reported that there is a cross-resistance association between oseltamivir and peramivir [49,69]. For patients infected with A/H1N1 virus harboring the H275Y mutation, studies conducted before the 2007-2009 seasons are therefore likely to underestimate resistance to peramivir dramatically. Furthermore, recent peramivir clinical trials have evaluated a parenteral injection form instead of prior oral tablets [28,70].

Most oseltamivir studies included a mix of population subgroups and had little or no reporting on subgroup results. As a result, much within-subgroup heterogeneity ensued, and thus, many of our subgroup analyses did not yield any 'apparent' differences. The presence of a mixture of resistant and susceptible viruses especially in immunocompromised hosts may also potentially complicate the interpretation of our findings. Lastly, we did not find any studies on incidence of antiviral resistance among H5N1 patients, and only two case reports on H5N1 patients [51,52]. As for case reports, it is likely that our search may have missed a substantial number of case reports due to such publications being poorly indexed in the utilized search engines. We excluded some case reports of oseltamivir resistance in H5N1 infected humans because no clinical data was reported [71] or because resistance was detected pre-therapy [72].

The rates at which pneumonia occurred in oseltamivir-susceptible patients were between 1% and 3%[45-48]. Meanwhile, our meta-analysis suggests that oseltamivir-resistant patients are 4-times more likely to suffer from pneumonia. Assuming that 2% of oseltamivir-susceptible patients suffer from pneumonia, 1 out of every 50 oseltamivir-susceptible patients would suffer from pneumonia in comparison to 1 out of every 12 oseltamivir-resistant patients. It is noteworthy that in the identified case reports, many cases of resistance arose in immunocompromised patients adding a confounder in the interpretation of this association.

Our review provides valuable insight on the incidence of antiviral resistance associated with oseltamivir use. However, we were not able to draw inferences about incidence of antiviral resistance associated with zanamivir or peramivir use due to the absence of recent studies on the topic. The reviewed case reports mainly involved immunocompromised subjects who were hospitalized (in-patients) and received oseltamivir as treatment rather than prophylaxis. This could suggest a tendency that notable complications associated with antiviral resistance more frequently arise among such patients. To confirm this, however, future cohort studies that investigate the associations between antiviral resistance and complications should incorporate patient and treatment characteristics in the multivariate models.

## Conclusion

Our results demonstrate that that a substantial number of patients may become oseltamivir-resistant as a result of oseltamivir use, and that oseltamivir resistance may be significantly associated with pneumonia. In contrast, zanamivir resistance has been rarely reported to date.

## Financial Competing interests

This review was funded by GlaxoSmithKline Inc. (GSK).

Lehana Thabane (LT) has received consultancies from several pharmaceutical companies including GSK, AstraZeneca, CanReg Inc., F. Hoffman La Roche, Theralase Inc, Sorono Canada Inc, Merck Frosst - Schering Pharmaceuticals, Pfizer, Proctor and Gamble Pharmaceuticals, and law firms that include Newton Wong and Associates, and Paterson McDougall.

Guy Boivin (BG) has received research grants and honoraria from GSK and Hoffman La Roche.

Tahany Awad (TA) has received consultancies from Hoffman La Roche.

Kristian Thorlund (KT) has no financial interests to declare.

## Non-financial Competing interests

The authors declare that they have no competing interests.

## Authors' contributions

KT drafted the protocol and the manuscript, assessed eligibility of identified study abstracts, extracted data, scored the quality of all eligible studies and performed all statistical analyses. TA contributed to the writing of the protocol and the manuscript, contributed to the design of the review, contributed to the interpretation of data, assessed eligibility of identified study abstracts, extracted data and scored the quality of all eligible studies. GB and LT contributed the writing of the protocol and manuscript, contributed to the design of the review and contributed to the interpretation of data.

All authors read and approved the final version of the manuscript.

## Pre-publication history

The pre-publication history for this paper can be accessed here:

http://www.biomedcentral.com/1471-2334/11/134/prepub

## Supplementary Material

Additional file 1 **Additional figure 1 (Figure A.1)**. Forest plots of antiviral resistance incidence among oseltamivir studies subgrouped by study design. The numerator is the number of patients who developed resistance, and the denominator is the number of patients that received an NAI. Click here for file

Additional file 2 **Additional figure 2 (Figure A.2)**. Forest plots of antiviral resistance incidence among oseltamivir studies subgrouped by immunocompetent and immunocompromised patients. The numerator is the number of patients who developed resistance, and the denominator is the number of patients that received an NAI. Click here for file

Additional file 3 **Additional figure 3 (Figure A.3)**. Forest plots of antiviral resistance incidence among oseltamivir studies subgrouped by out-patients and in-patients. The numerator is the number of patients who developed resistance, and the denominator is the number of patients that received an NAI. Click here for file

Additional file 4 **Additional figure 4 (Figure A.4)**. Forest plots of antiviral resistance incidence among oseltamivir studies subgrouped by study age group (adults or children). The numerator is the number of patients who developed resistance, and the denominator is the number of patients that received an NAI. Click here for file

Additional file 5 **Additional figure 5 (Figure A.5)**. Forest plots of antiviral resistance incidence among oseltamivir studies subgrouped by intervention purpose. The numerator is the number of patients who developed resistance, and the denominator is the number of patients that received an NAI. Click here for file

Additional file 6 **Additional figure 6 (Figure A.6)**. Forest plots of risk ratios for associations between antiviral resistance and clinical symptoms. All risk ratio estimates are crude estimates Click here for file

Additional file 7 **Additional figure 6 (Figure A.6)**. Forest plots of risk ratios for associations between antiviral resistance and clinical symptoms. All risk ratio estimates are crude estimates Click here for file
