# Supplementary figures and images for: Systematic review of influenza resistance to the neuraminidase inhibitors
Source: BMC Infect Dis. 2011 May 19;11:134. doi: 10.1186/1471-2334-11-134 (PMC3123567; doi:10.1186/1471-2334-11-134)

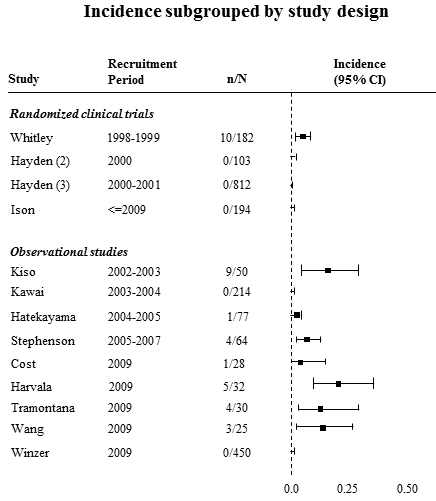

Supplement: Additional file 1 — Additional figure 1 (Figure A.1). Forest plots of antiviral resistance incidence among oseltamivir studies subgrouped by study design. The numerator is the number of patients who developed resistance, and the denominator is the number of patients that received an NAI. [file 1471-2334-11-134-S1.TIFF]

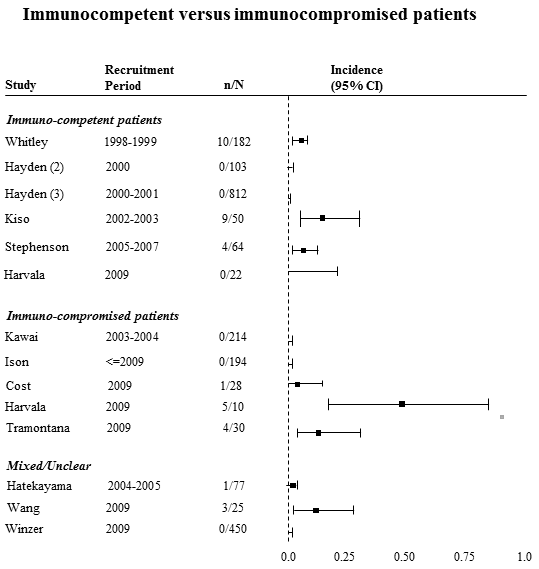

Supplement: Additional file 2 — Additional figure 2 (Figure A.2). Forest plots of antiviral resistance incidence among oseltamivir studies subgrouped by immunocompetent and immunocompromised patients. The numerator is the number of patients who developed resistance, and the denominator is the number of patients that received an NAI. [file 1471-2334-11-134-S2.TIFF]

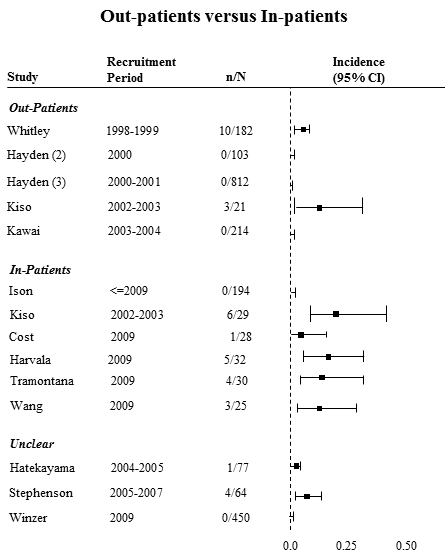

Supplement: Additional file 3 — Additional figure 3 (Figure A.3). Forest plots of antiviral resistance incidence among oseltamivir studies subgrouped by out-patients and in-patients. The numerator is the number of patients who developed resistance, and the denominator is the number of patients that received an NAI. [file 1471-2334-11-134-S3.TIFF]

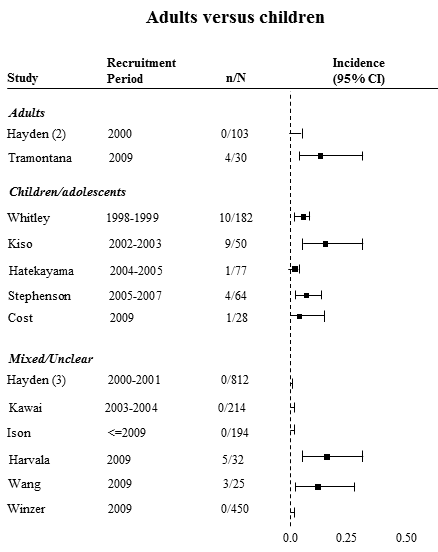

Supplement: Additional file 4 — Additional figure 4 (Figure A.4). Forest plots of antiviral resistance incidence among oseltamivir studies subgrouped by study age group (adults or children). The numerator is the number of patients who developed resistance, and the denominator is the number of patients that received an NAI. [file 1471-2334-11-134-S4.TIFF]

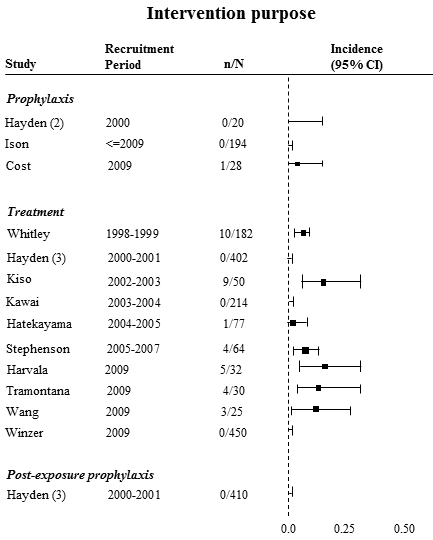

Supplement: Additional file 5 — Additional figure 5 (Figure A.5). Forest plots of antiviral resistance incidence among oseltamivir studies subgrouped by intervention purpose. The numerator is the number of patients who developed resistance, and the denominator is the number of patients that received an NAI. [file 1471-2334-11-134-S5.TIFF]

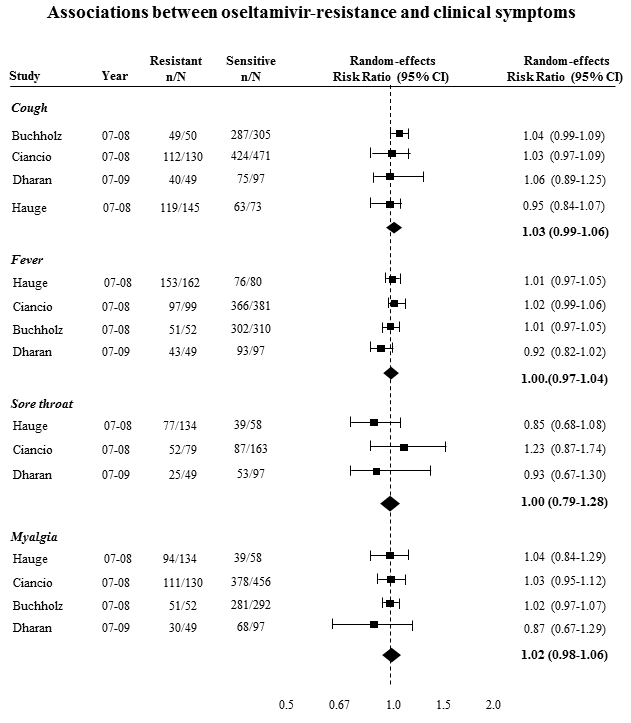

Supplement: Additional file 6 — Additional figure 6 (Figure A.6). Forest plots of risk ratios for associations between antiviral resistance and clinical symptoms. All risk ratio estimates are crude estimates [file 1471-2334-11-134-S6.TIFF]

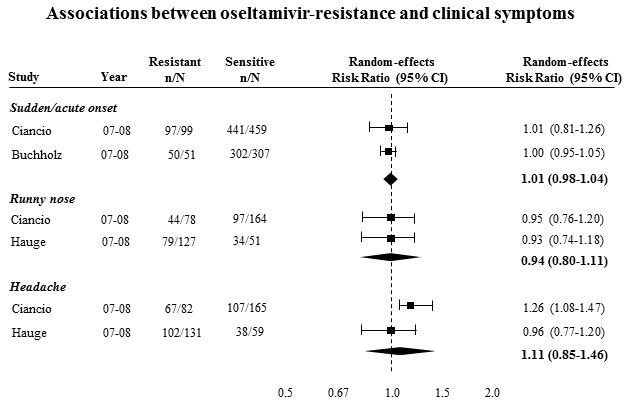

Supplement: Additional file 7 — Additional figure 6 (Figure A.6). Forest plots of risk ratios for associations between antiviral resistance and clinical symptoms. All risk ratio estimates are crude estimates [file 1471-2334-11-134-S7.TIFF]
